# Noncollinear Magnetic Order in Two-Dimensional NiBr_2_ Films Grown on Au(111)

**DOI:** 10.1021/acsnano.1c05221

**Published:** 2021-09-07

**Authors:** Djuro Bikaljević, Carmen González-Orellana, Marina Peña-Díaz, Dominik Steiner, Jan Dreiser, Pierluigi Gargiani, Michael Foerster, Miguel Ángel Niño, Lucía Aballe, Sandra Ruiz-Gomez, Niklas Friedrich, Jeremy Hieulle, Li Jingcheng, Maxim Ilyn, Celia Rogero, José Ignacio Pascual

**Affiliations:** † CIC nanoGUNE-BRTA, 20018 Donostia-San Sebastián, Spain; ‡ Institute of Physical Chemistry, University of Innsbruck, Innrain 52c, A-6020 Innsbruck, Austria; ¶ Centro de Física de Materiales (CSIC/UPV-EHU), 20018 Donostia-San Sebastián, Spain; § Paul Scherrer Institut, Forschungsstrasse 111, CH-5232 Villigen, PSI, Switzerland; ∥ ALBA Synchrotron Light Source, Carrer de la Llum, 2-26, 08290 Barcelona, Spain; ⊥ Donostia International Physics Center DIPC, 20018 Donostia-San Sebastián, Spain; # Ikerbasque, Basque Foundation for Science, 48013 Bilbao, Spain

**Keywords:** 2D metal dihalide, molecular beam epitaxy, van der Waals material, semiconductor, 2D magnetism

## Abstract

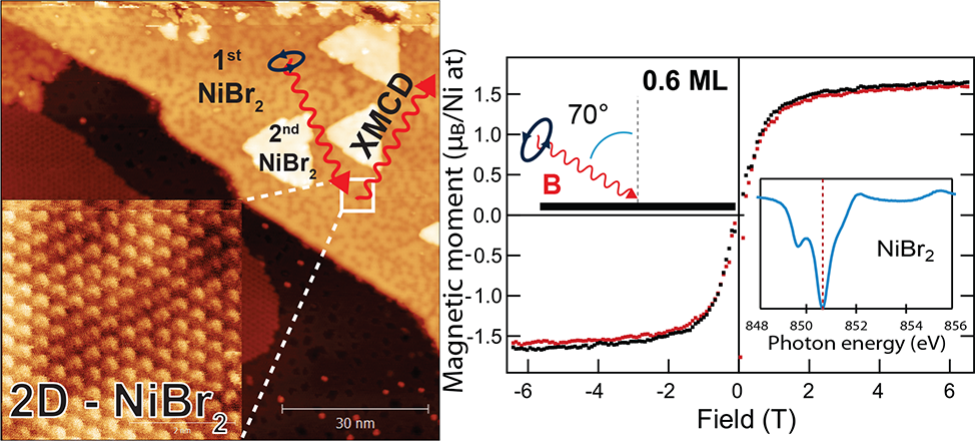

Metal halides are a class of layered materials with promising electronic and magnetic properties persisting down to the two-dimensional limit. While most recent studies focused on the trihalide components of this family, the rather unexplored metal dihalides are also van der Waals layered systems with distinctive magnetic properties. Here we show that the dihalide NiBr_2_ grows epitaxially on a Au(111) substrate and exhibits semiconducting and magnetic behavior starting from a single layer. Through a combination of a low-temperature scanning-tunneling microscopy, low-energy electron diffraction, X-ray photoelectron spectroscopy, and photoemission electron microscopy, we identify two competing layer structures of NiBr_2_ coexisting at the interface and a stoichiometrically pure layer-by-layer growth beyond. Interestingly, X-ray absorption spectroscopy measurements revealed a magnetically ordered state below 27 K with in-plane magnetic anisotropy and zero-remanence in the single layer of NiBr_2_/Au(111), which we attribute to a noncollinear magnetic structure. The combination of such two-dimensional magnetic order with the semiconducting behavior down to the 2D limit offers the attractive perspective of using these films as ultrathin crystalline barriers in tunneling junctions and low-dimensional devices.

Since the discovery of graphene in 2004, research on ultrathin 2D films has become a subject of tremendous interest.^1,2^ The number of 2D materials is growing rapidly, including metals, semimetals, semiconductors, insulators, and superconductors.^3−7^ By decreasing the dimensionality of the bulk material down to the 2D limit, fascinating physical and chemical properties appear, which offer great possibilities for low-dimensional applications,^8−12^ but bear as well huge potential for the exploration of exotic physical phenomena by means of constructing van der Waals heterostructures.^4,13^ Furthermore, it is foreseen that 2D materials can substitute silicon in the complementary metal–oxide–semiconductor switches, in order to provide lower power consumption, thus delivering a higher performance.^14^ The prospect of using 2D materials in electronics requires investigation of their properties both on the microscopic and mesoscopic level, reaching the length scale relevant for possible low-dimensional applications.

Magnetic 2D materials are of special interest for fabricating superconductor–ferromagnetic van der Waals interfaces,^7,15^ but also for ultrathin barriers in tunneling devices.^16,17^ However, two-dimensional intrinsic ferromagnetic (FM) materials are still in their emergence.^2^ In order to realize a ferromagnetic 2D system, one has to overcome the constraints of the Mermin–Wagner theorem,^18^ where long-range magnetic order is suppressed due to thermal fluctuations.^19,20^ This can be achieved by, for example, applying a magnetic field or mechanical stress onto the system but also—more interestingly—by intrinsic magnetic properties of the material as a consequence of strong spin–orbit coupling (SOC).^18,19^

There are plenty of theoretical studies predicting magnetic monolayer materials,^21−28^ but only a few intrinsic 2D ferromagnets were experimentally found.^7,20,29−37^ The most explored magnetic 2D materials are trivalent halides of the type MX_3_ (M = metal, X = halogen) .^7,29−31,37^ Bulk divalent halides (MX_2_) are known to be magnetic, but they still have not been studied thoroughly in the limit of a single layer.^38,39^ A promising candidate that belongs to this family and was predicted to possess intrinsic ferromagnetism down to the 2D limit is NiBr_2_.^21,22,40,41^

Bulk NiBr_2_ is a van der Waals semiconducting material with an interlayer spacing of 3.24 Å.^21^ This large separation implies a weak interlayer coupling and hints at the possibility of exfoliation into single-layer sheets. A single, pristine layer of NiBr_2_ consists of a Ni plane embedded between two Br planes with an in-plane lattice constant of 3.7 Å.^21,22^ The nickel ion is in a +2 oxidation state after using two of their valence electrons for ionic bonding with bromine. The octahedral coordination of the nickel ion splits the electronic states of the d-shell into two groups of levels with t_2g_ and e_g_ symmetry. The first group of levels is full with six electrons, while the second is half-filled, endowing the Ni^2+^ ion with a nominal magnetic moment of 2 μ_B_.^38^ The ordered magnetic state observed in bulk NiBr_2_ below 52 K comprises ferromagnetic slabs stacked antiferromagnetically along the crystallographic *c*-axis.^38^ Calculations on bidimensional NiBr_2_ suggest a ferromagnetic ground state with a net magnetic moment between 1.57 and 1.88 μ_B_.^21,22^ The predicted value of the Curie temperature (*T*_C_) for a monolayer of NiBr_2_ is 136–140 K, which is rather high as compared to 2D trivalent halides (*T*_C_ < 45 K).^21,22^

In this work we show that NiBr_2_ grows epitaxially on a Au(111) substrate and exhibits semiconducting character and magnetic ordering behavior already from the first monolayer. To determine the growth modes and their chemical stoichiometry, we combined diverse surface analytical methods such as low-temperature scanning-tunneling microscopy (STM) and spectroscopy (STS), low-energy electron diffraction (LEED) and microscopy (LEEM), X-ray photoemission (XPS), and photoemission electron microscopy (PEEM). We find that the layer-by-layer growth of NiBr_2_ proceeds in the form of weakly coupled layers, with the exception of partly decomposed NiBr_2_ domains at the interface, probably caused by the catalytic effect of the gold substrate. X-ray magnetic circular dichroism (XMCD) measurements unveiled a magnetically ordered state below 27 K with in-plane magnetic anisotropy and zero remanence in the single NiBr_2_ layer that we interpret as a noncollinear magnetic structure.

## Results and Discussion

### Growth and Structure of NiBr_2_ on Au(111)

**Figure 1:**
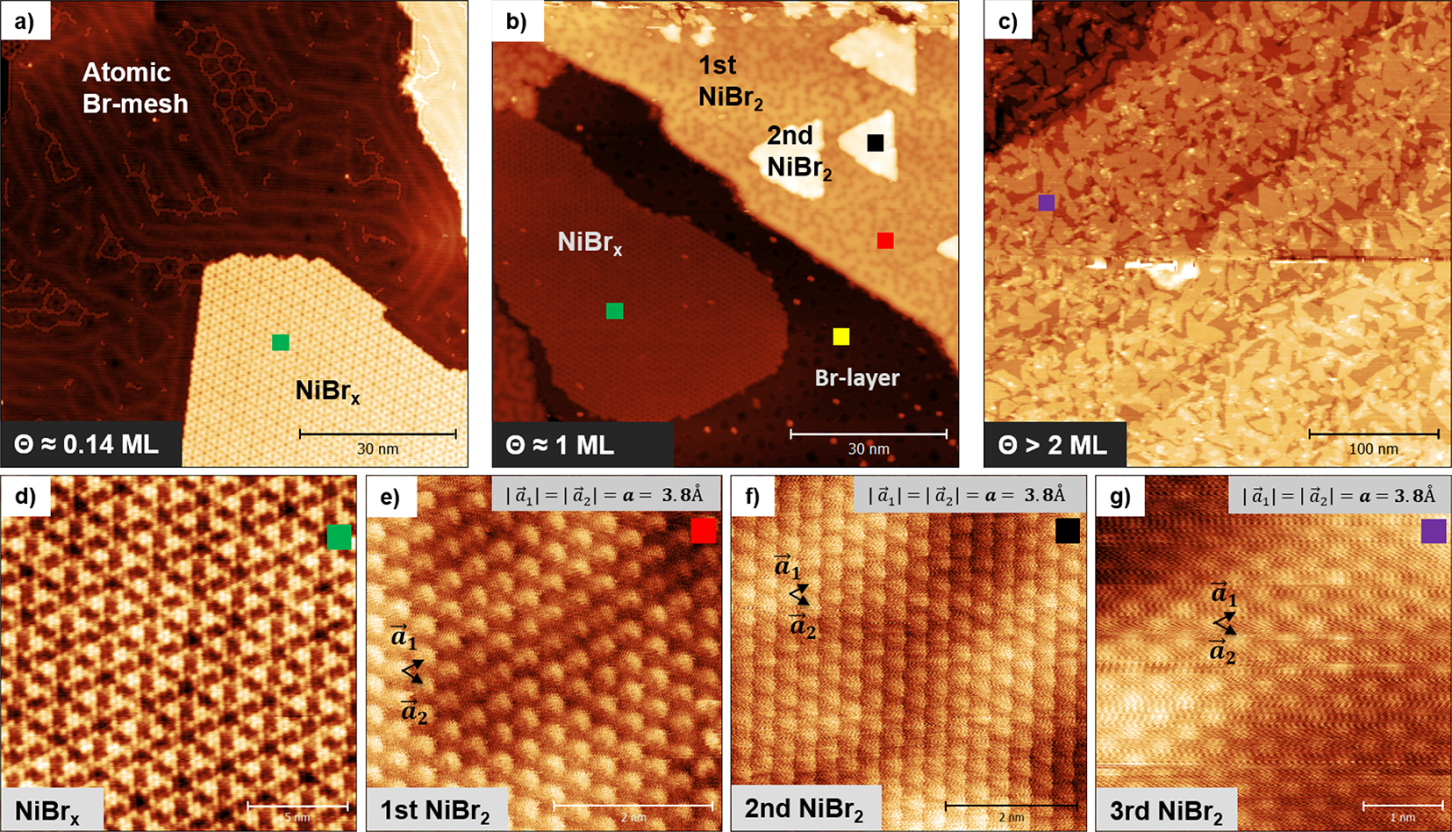
STM images of different coverages of NiBr_2_ on Au(111) and the corresponding layer structure. (a) Submonolayer coverage with islands of NiBr_*x*_ (labeled with a green rectangle) and distributed Br atoms on the surface. *I* = 60 pA, *U* = 1 V. (b) Coexistence of three different first layers (green, yellow, red rectangles) and second-layer islands (black rectangle). *I* = 100 pA, *U* = 1 V. (c) NiBr_2_ multilayer. *I* = 30 pA, *U* = 1 V. (d) Atomic layer structure of NiBr_*x*_ (green rectangle). *I* = 20 pA, *U* = 1 V, scale bar: 5 nm. (e–g) Atomic resolution of the first (red rectangle), second (black rectangle), and third (violet rectangle) pristine layer of NiBr_2_, showing the same lattice parameters: (e) *I* = 4.7 nA, *U* = 0.01 V, scale bar: 2 nm; (f) *I* = 1.8 nA, *U* = 0.05 V, scale bar: 2 nm; (g) *I* = 100 pA, *U* = −1.6 V, scale bar: 1 nm.

**Figure 2:**
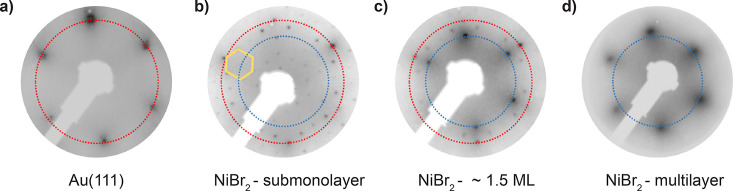
LEED pattern for different coverages of NiBr_2_ on Au(111). (a) Hexagonal pattern for the clean Au(111) surface. (b) LEED pattern representing the sub-monolayer regime with the majority of NiBr_*x*_. (c) LEED image showing ∼1.5 ML NiBr_2_. Spots characteristic of NiBr_2_ and indicated by the blue circle are more pronounced than in (b). (d) Hexagonal pattern showing the crystal structure of pristine NiBr_2_. All LEED images have been collected at 137 eV.

To investigate the growth mode of NiBr_2_, we compare in Figure 1 low-temperature STM images of the substrate with different NiBr_2_ coverages. High-resolution STM images of every different domain are shown in Figure 1d–g to identify their layer structure and interpret their origin. For a nominal coverage of ∼0.1 ML, highly ordered two-dimensional islands appear coexisting with atomic chains distributed along the Au(111) surface (Figure 1a). We attribute these to residual Br atoms forming a mesh on the gold surface.^42^ According to thermochemical investigations, sublimation of NiBr_2_ produces a gas of monomers and dimers of stoichiometric NiBr_2_, while a thermal decomposition of the precursor is negligible.^43^ However, the catalytic activity of the Au(111) surface in the dehalogenation reaction leads to decreasing energy barriers for dissociation of halogen atoms.^44^ Therefore, the presence of residual Br atoms is most probably a result of the dissociative adsorption of some part of the NiBr_2_ molecules.^45^ Further, the Au(111) reconstruction seems to be slightly modified, suggesting a chemical interaction of the NiBr_*x*_ species with the metal substrate. The internal layer structure of the NiBr_*x*_ island, depicted in Figure 1d, appears with a complex chiral structure, with atomic periodicity of ∼3.6 Å, and with an additional superstructure of ∼11.9 Å (these can be extracted from the fast Fourier transformation (FFT) analysis shown in the Supporting Information (SI), in Figure S1). This layer structure is further confirmed by its distinctive LEED pattern, displayed in Figure 2b. As indicated by the red and blue circles, the unit cell of the NiBr_*x*_ overlayer is a factor 4/3 larger than the Au(111) surface, resulting in a 4 × 4 superstructure with respect to the underlying Au(111) (note that the lattice constant of the NiBr_*x*_ layer, 3.6 Å, is close to a factor 4/3 of the Au(111) lattice parameter, 2.87 Å). Thus, we suggest that the NiBr_*x*_ phase is composed of a Au-commensurate Ni-plane with chemically bound Br atoms on top.

Stoichiometic NiBr_2_ layers can also be observed directly on the Au(111) surface, especially when increasing the coverage. Deposition of a ∼1 ML of NiBr_2_ leads to the appearance of additional hexagonal regions (red square in Figure 1b) with a lattice constant of 3.8 Å (Figure 1e). We attribute this layer to intact NiBr_2_ domains, since their structure and lattice periodicity coincide with that of the nominal value for a free two-dimensional NiBr_2_ layer (with lattice constant 3.7 Å, according to DFT simulations^21,22^). It is noteworthy that the first NiBr_2_ layer appears with some contrast formed by brighter and darker patches, probably caused by interaction with the underlying Au substrate. The STM images for this coverage also show some second-layer nucleation on top of the first NiBr_2_ layer, with the same bulk-like NiBr_2_ periodicity (black rectangle, see Figure 1b,f). The second layer does not show any prominent bright–dark superstructure. Figure 2c represents a LEED pattern for the ∼1.5 ML sample. It is similar to the pattern in Figure 2b. However, the spots indicated by the inner blue circle are more intense, suggesting a coexistence of NiBr_*x*_ and NiBr_2_, with increasing proportion of the latter.

STM images of thicker NiBr_2_ films, with an estimated coverage above 2 ML, show the nucleation of a third NiBr_2_ layer on top of a complete second one (Figure 1c), also with the bulk lattice structure (Figure 1g), in agreement with a layer-by-layer growth mode. The LEED pattern of the multilayer film comprises only the diffuse hexagonal pattern and has no traces of the bare Au(111) pattern. The dimension of the hexagonal cell in Figure 2d (indicated by a blue circle) fits well with the unit cell of the bulk NiBr_2_ (3.8 Å). The absence of additional spots characteristic of the 4 × 4 superstructure emphasizes the growth of exclusively stoichiometric NiBr_2_ layers for higher coverages.

**Figure 3:**
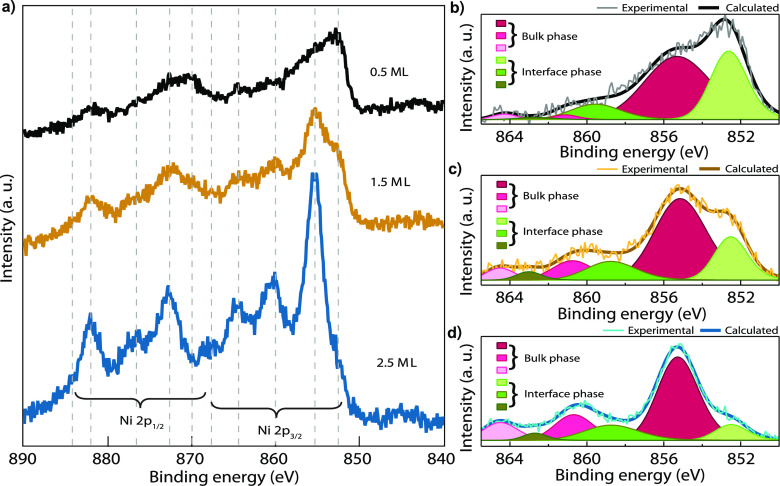
(a) XPS spectra showing the characteristic Ni 2p core level peaks for different coverages of NiBr_2_ on Au(111). Gray dashed lines demonstrate the position of the peaks. (b–d) Detailed analysis of the Ni 2p_3/2_ signal within the range of 850–865 eV, showing the proportional composition of the bulk (NiBr_2_) and the interface (NiBr_*x*_) phases.

The chemical composition of the epitaxial NiBr_2_ sheets has been probed using X-ray photoemission spectroscopy. Figure 3a shows the Ni 2p core level measured for coverages ranging from submonolayer to ∼2.5 ML. The two spin–orbit components (Ni 2p_3/2_ and Ni 2p_1/2_), separated by Δ ≈ 17.3 eV, exhibit a complex multiple-peak structure due to different core–hole final states and plasmon resonances.^46^ The spectra measured for 2.5 ML, blue in Figure 3a, reproduce the eight peak shape spectra reported for bulk NiBr_2_,^47^ indicating that the NiBr_2_ stoichiometric phase is indeed preserved after sublimation. For lower coverage, both the shape and position of the peaks change, revealing the presence of NiBr_*x*_ with a different Ni chemical environment. Figure 3b–d compare in detail the Ni 2p_3/2_ spectra for the three measured coverages together with their decomposition in components. Following the model of ref (46) we used three peaks (main peak located at ∼855.2 eV plus two satellites at higher B.E.) to fit the Ni 2p_3/2_ spectra of NiBr_2_ (red peaks in Figure 3b–d). We found out that a good description of the experimental spectra can be obtained if we keep their positions constant and assign another three peaks shifted ∼2.5 eV toward the lower binding energy to represent a contribution of the NiBr_*x*_ phase (green peaks in Figure 3b–d). All spectra were aligned and normalized using the Au 4d peak (not shown). Figure 3b–d clearly demonstrate quick attenuation of the NiBr_*x*_ signal with nucleation of the second and third layers of NiBr_2_ in the 1.5 and 2.5 ML samples, respectively. It corroborates that NiBr_*x*_ exists only in the Au interface. Furthermore, Figure 3b shows substantial contribution of the NiBr_2_ signal in the experimental spectrum of 0.5 ML sample, proving that the first layer comprises both phases. Thus, XPS data agree with the STM and LEED results.

### Layer-Dependent Electronic Band Gap

The electronic properties of ultrathin NiBr_2_ films on Au(111) were measured by means of d*I*/d*V* spectra at various surface positions.

**Figure 4:**
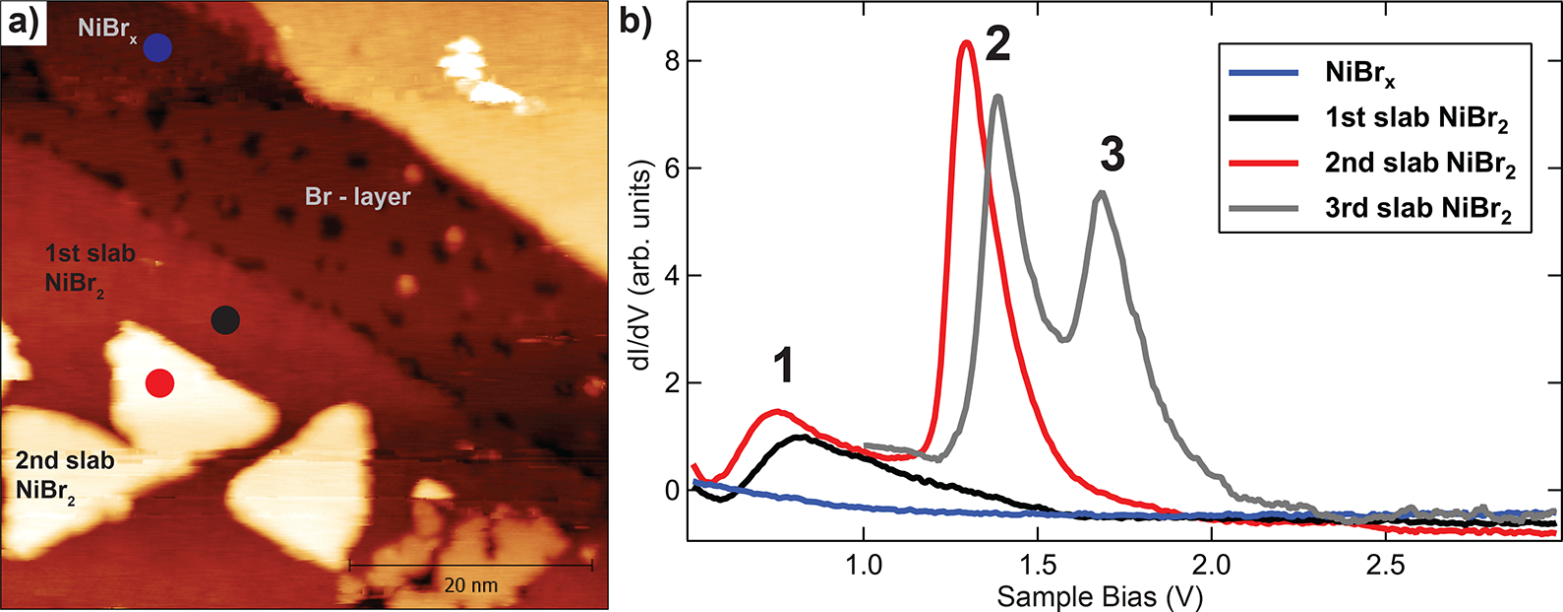
STS spectra measured on different surface layer structures, revealing corresponding resonances for positive sample bias (unoccupied states). (a) STM image with spectroscopy positions marked in different colors; *I* = 100 pA, *U* = 3 V. (b) Constant-current d*I*/d*V* spectra measured on the different points indicated in (a) and corresponding to different layer structures (current set point *I* = 100 pA).

Figure 4a shows an STM image with a nominal coverage of 1 ML NiBr_2_ on the Au(111) surface. We study first the unoccupied states of the different halide phases and layers. Constant-current d*I*/d*V* spectra measured at the positions marked as colored circles in the STM image are displayed in Figure 4b. The spectrum on the NiBr_*x*_ interface phase (blue in Figure 4b) does not show any distinct features in the d*I*/d*V* signal. It is also noteworthy that the Au(111) Shockley surface state at *ca*. −0.5 V does not survive upon deposition of NiBr_*x*_ (see Figure S2 in the SI). A rather metallic character can be observed that agrees with a partial debrominated phase and with the larger hybridization of Ni ions with the metal substrate. In contrast, spectra on NiBr_2_ layers show sharp resonances for the unoccupied density of states, whose positions depend on the number of layers: the first NiBr_2_ layer (black spectrum in Figure 4b) shows a single, broad resonance at ∼0.8 V (peak 1); the spectrum on the second layer (red) shows an additional resonance peak at ∼1.3 V (peak 2), while the third layer (gray) shows two additional resonances, one attributed to peak 2 shifted to ∼1.4 V and an additional peak 3, at ∼1.7 V.

**Figure 5:**
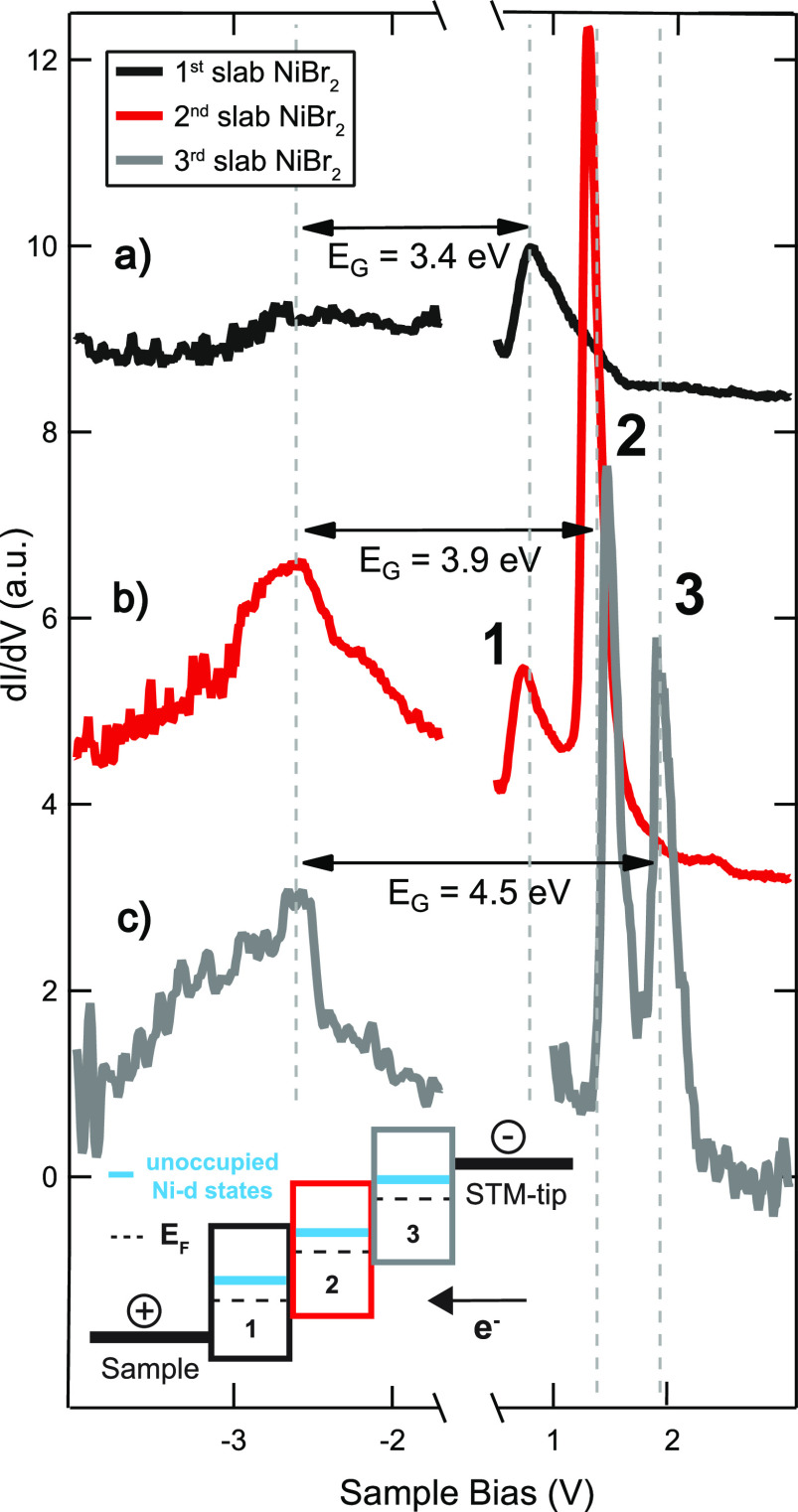
Determination of the band gap for different numbers of NiBr_2_ layers: (a) first slab NiBr_2_, (b) second slab NiBr_2_, (c) third slab NiBr_2_. The spectrum on the third layer (gray) was measured on a different preparation, as in Figure 1c. Inset: Schematics of the tunneling process with respect to the unoccupied states through the layers of NiBr_2_.

Band structure calculations for a free NiBr_2_ layer found that the material is a semiconductor with a band gap of 4 eV^21^ and a characteristic unoccupied flat band above *E*_F_, attributed to the largely localized Ni d orbitals. The sharp d*I*/d*V* resonances obtained for the different layers in Figure 4b agree in energy alignment and shape with these frontier flat bands. Interestingly, we observe an increasing number of resonances with the number of layers. We interpret these as different resonant tunneling processes, each through the Ni-d flat band of the respective stacked NiBr_2_ layers. Due to the weak van der Waals interaction between each layer, the Ni-d bands are highly localized at the respective Ni position and very weakly coupled to neighboring layers, so they can also be aligned with different energy. We expect that the increasing separation of every layer from the gold substrate leads to a decreasing screening effect by the metal substrate. This causes the flat bands of higher layers to appear shifted to higher energy in the spectrum, thus leading to a larger effective band gap. In addition, we note that for such layered system the applied potential difference between tip and sample during the STS measurement may cause electrostatic shifts in each isolated Ni-d band (*i.e*., local gating) depending on their position in the junction (a schematic model of the tunneling process is depicted in the inset of Figure 5).

To obtain the values of the effective band gaps for every layer, we compare in Figure 5 constant-current d*I*/d*V* spectra on the first, second, and third slab of the pristine NiBr_2_, including spectra of the occupied states (an equivalent constant-height d*I*/d*V* spectrum, showing the fully depleted DOS within the energy gap, can be found in the SI in Figure S3). All layers show an increase in the d*I*/d*V* signal at *ca*. −2.6 V that we ascribe to the onset of the valence band (VB) of the NiBr_2_ film. In contrast to the strongly localized flat Ni-d states for the unoccupied states, the weak and rather broad resonance peak at *ca*. −2.6 V is attributed to the existence of overlapping s, p, and d bands with a major contribution from the Br-4p orbitals.^21,22^ Attributing the different resonance peaks at positive sample biases to the respective conduction band (CB) onsets, the data reveal that the semiconducting behavior persists for each layer. The separation between the CB and VB peak(s) of the first layer of NiBr_2_ amounts to *E*_G_ = 3.4 (±0.2) eV and increases to *E*_G_ = 3.9 (±0.2) eV and *E*_G_ = 4.5 (±0.2) eV for the second and third layer, suggesting an increasing effective gap of the system. This is probably due to both a gradually smaller screening effect of the metal substrate and a small local gating of the unoccupied bands during the STS measurement. Nevertheless, these values lie close to the gap obtained from first-principle calculations of 4.0 eV.^22^

### Magnetic Properties

**Figure 6:**
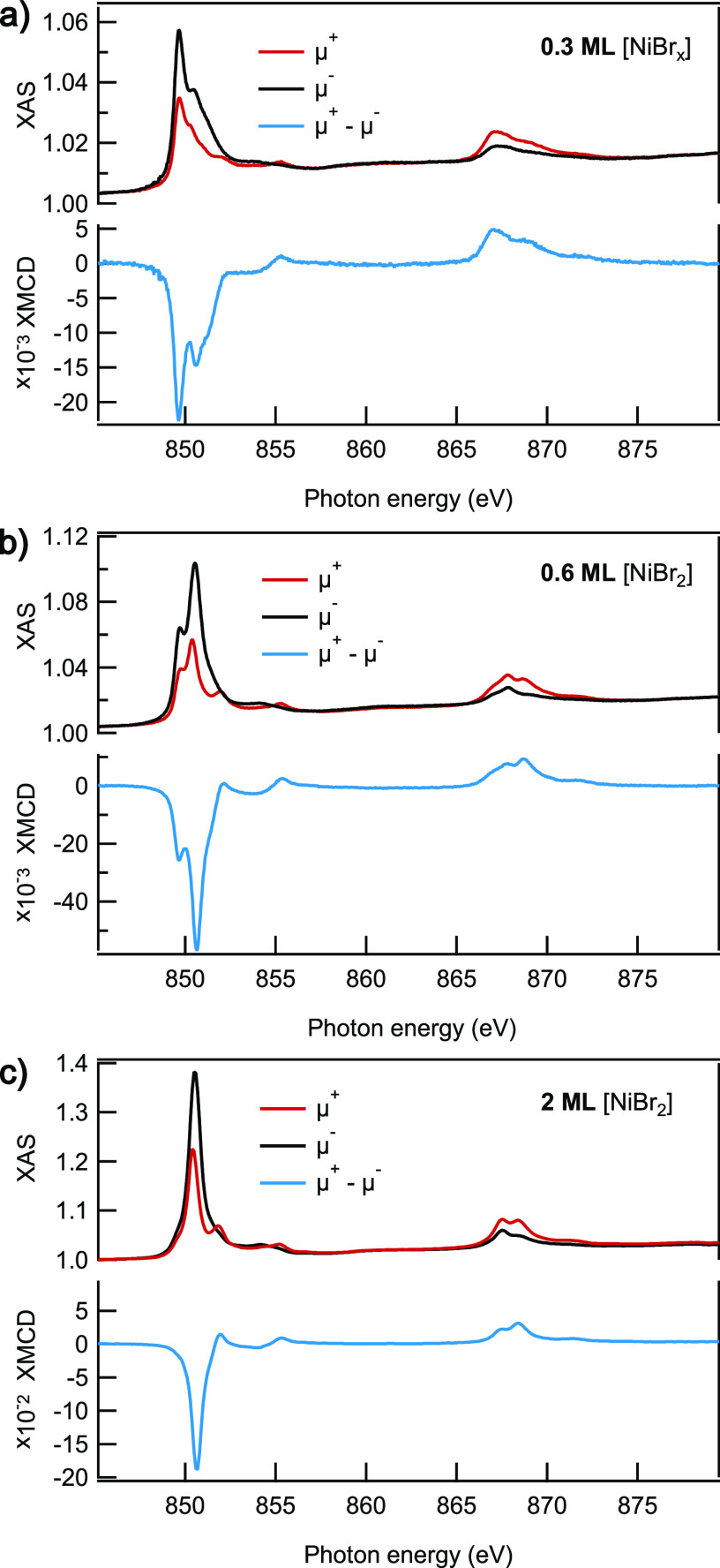
XAS and respective XMCD spectra taken with circular right (black) and circular left (red) polarization in a field of 6.5 T and at a temperature of 3 K (a, b) and of 6 T at 3.5 K (c). All data were collected in grazing incidence geometry (70°). It is worth mentioning that positions of the peaks in XAS and XPS spectroscopy do not coincide because Ni L_3,2_ absorption edges correspond to the transition from Ni 2p to the Ni 3d state, while XPS emission from the Ni 2p state measures transitions to the continuum.

To resolve the possible survival of a magnetic state in two-dimensional NiBr_2_ layers, magnetic measurements were performed by means of the spatially averaging XMCD technique. XAS spectra acquired for three samples with different dihalide coverage are shown in Figure 6 together with the resulting XMCD spectra. Peaks of the XAS absorption at the Ni L_3_ edge have two components that give rise to two distinctive maxima in the XMCD spectra close to 850 eV. Comparing these data with XPS spectra from Figure 3, we can attribute the component with an absorption peak below 850 eV with a NiBr_*x*_ phase that has a lower binding energy of the respective Ni 2p_3/2_ state and the second component, with an absorption peak above 850 eV, with the NiBr_2_ phase. Evolution of the XAS spectra with coverage follows the trend observed with XPS: a 0.3 ML sample contains more NiBr_*x*_ than NiBr_2_, but in the 0.6 ML sample the stoichiometric NiBr_2_ already prevails over the NiBr_*x*_ phase. Eventually, in the XAS spectra acquired for the 2 ML sample the contribution of the NiBr_*x*_ phase is negligible in comparison to the strong signal corresponding to the NiBr_2_ (see Figure 6c).

As mentioned before, the eight d electrons of the octahedrally coordinated divalent Ni-ion in bulk NiBr_2_ occupy the six available states in the t_2g_ orbitals completely and half fill the e_g_ ones, giving rise to the configuration with a full spin *S* = 1 and with zero orbital moment.^38^ Nevertheless, for the samples with a sub-monolayer amount of NiBr_2_ on Au(111) the orbital magnetic moment calculated *via* sum rules reaches 0.34–0.41 μ_B_ per Ni atom and its spin magnetic moment ascends only to 1.27–1.49 μ_B_, which is substantially lower than 2 μ_B_, which would be the nominal value for the system with spin *S* = 1 (see Table S1) and discussion therein about the assumptions that were made to perform the analysis of the multicomponent system). Since the XAS spectra measured over Ni L_3,2_ absorption edges contain the contributions from both NiBr_*x*_ and NiBr_2_, analysis of the respective XMCD spectra *via* sum rules yields the values of the moments that are the weighted average of both components. Therefore, results for the samples with 0.3–1.0 ML coverage that have a notable amount of NiBr_*x*_ show that the magnetic properties of the decomposed phase are also rather different from stoichiometric NiBr_2_, in line with the striking difference demonstrated above for the electronic properties. In contrast, the 2 ML sample with a higher relative amount of NiBr_2_ possesses a lower average orbital magnetic moment of 0.11 μ_B_ and spin magnetic moment of 1.47 μ_B_, revealing that the magnetic properties of the first and second layers of the NiBr_2_ on Au(111) are approaching the properties of bulk NiBr_2_.^38^

**Figure 7:**
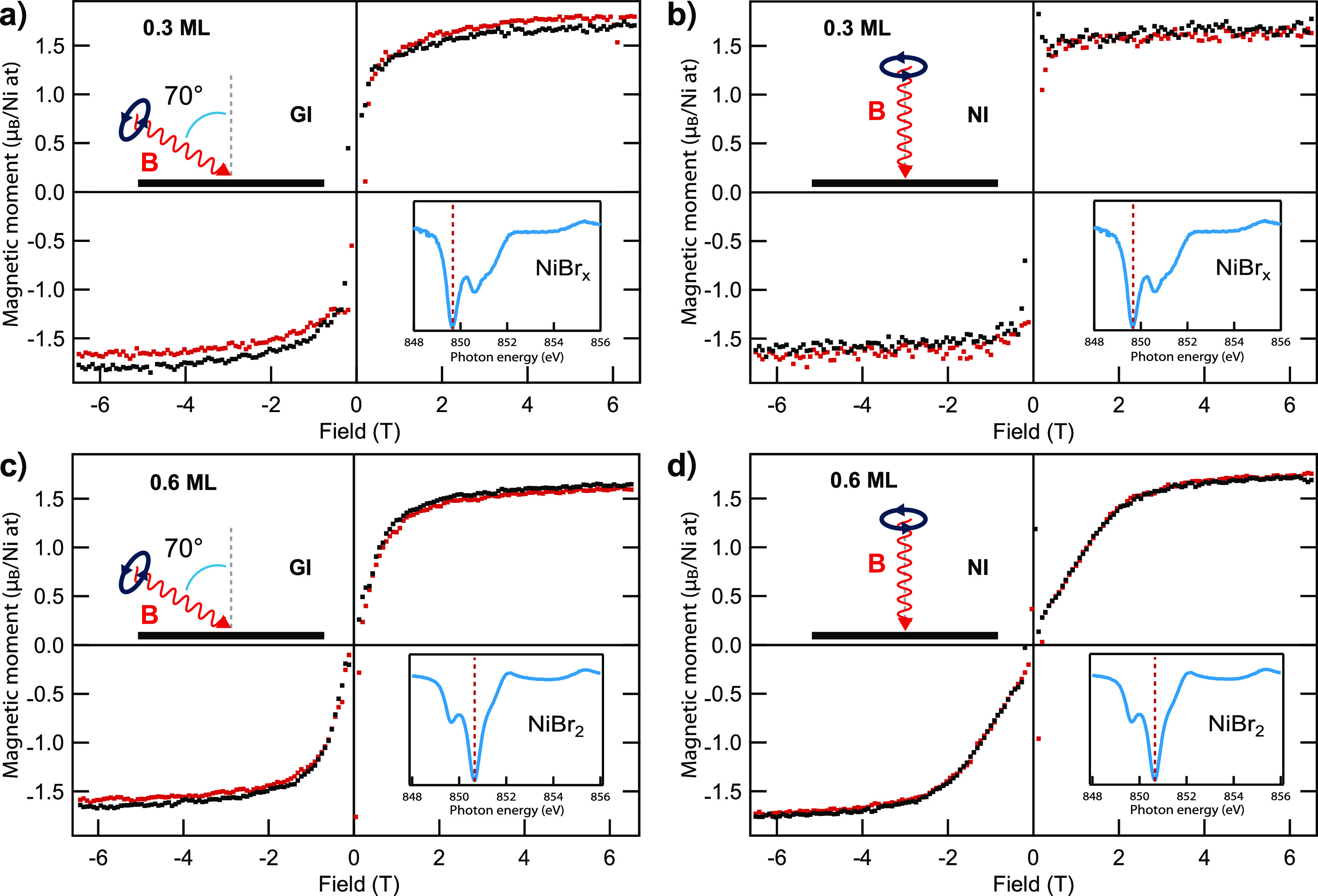
Comparison of magnetization loops measured in the grazing (70°) and normal incidence at the Ni L_3_ edge for the sample having a 0.3 ML coverage (majority of NiBr_*x*_ phase) (a, b) and 0.6 ML coverage (majority of NiBr_2_ phase) (c, d) at 3 K. Black and red points correspond to descending and ascending branches, respectively. NI and GI stands for normal incidence and grazing incidence, respectively.

XMCD magnetization loops were measured tracking the variation of the highest peak of the XMCD signal at the L_3_ Ni edge as a function of the applied magnetic field. Figure 7a,b show two of these curves acquired for the 0.3 ML sample at 3 K in grazing (GI) and normal incidence (NI), respectively. Measurements of this kind are not capable of assessing the magnetic moment close to zero field, yielding the artifacts within ±0.1 T, and therefore do not capture the hysteresis with the coercive force falling within this range. Nevertheless, they reveal that in the NI geometry magnetization remains almost constant. Further analysis provided in the SI proves the presence of nonzero remanence that is not concerned with magnetic anisotropy but originates from the magnetic order. Such a low-temperature ferromagnetic phase of NiBr_*x*_ is confirmed by inspecting the corresponding Arrott plots shown in the SI. Furthermore, since the low-field magnetization is almost equal to the saturation magnetization in NI geometry, such ferromagnetic phase has an out-of-plane easy magnetization direction (OOP anisotropy).

Magnetization loops measured for the 0.6 ML sample (Figure 7c,d) show that the magnetization approaches zero in low magnetic fields for both orientations. Nonhysteretic magnetization reversal and a lack of remanent magnetization allow us to discard collinear ferromagnetic order down to 3 K in the monolayer-thick NiBr_2_, which is the majority phase in the 0.6 ML sample. Nevertheless, these curves do not show the Curie–Weiss behavior expected for a simple paramagnet. Indeed, a saturation field for NI is almost twice as high as for the GI direction. The loops have no characteristic S-shape, but, instead, the magnetization increases almost linearly until saturation (a small nonlinear contribution close to zero field can be attributed to the minor amount of a NiBr_*x*_ phase present in this sample).

**Figure 8:**
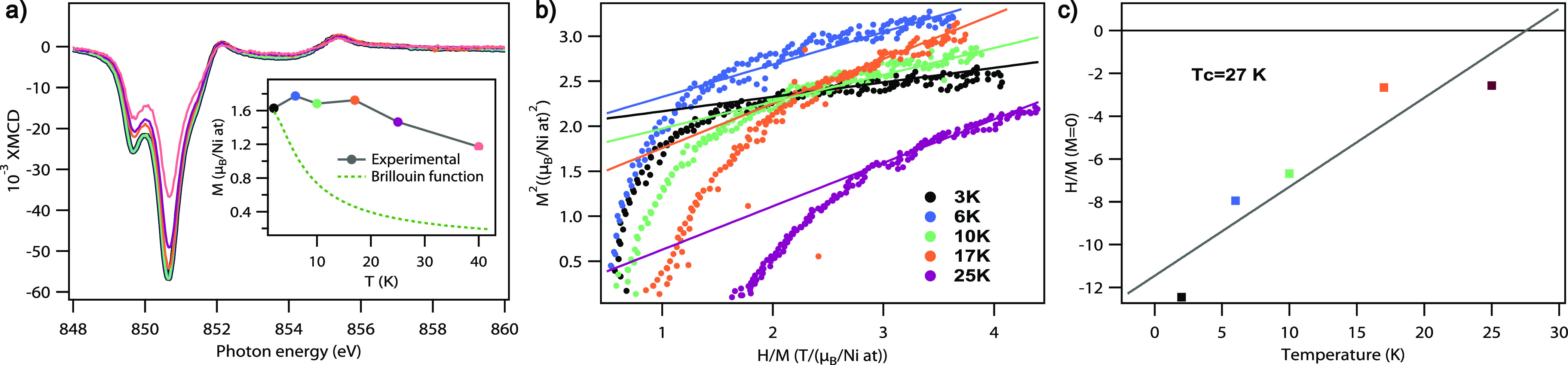
(a) XMCD spectra measured over the Ni L_3_ edge for the 0.6 ML sample at GI in the field of 6.5 T at different temperatures. The inset shows the in-plane saturation magnetization obtained *via* sum rules analysis and its behavior expected for the paramagnetic sample obeying the Brillouin function. (b) Arrott plot made for the 0.6 ML sample, having a majority of monolayer-thick NiBr_2_ phase and (c) respective intercept values.

Furthermore, sum rules analysis of the XMCD spectra collected at different temperatures (Figure 8a) allowed us to identify the evolution of the in-plane saturation magnetization. The experimental curve shown in the inset of Figure 8a displays only a slightly descending tendency in contrast with a quick decay predicted by the Brillouin function for the paramagnetic system.^48^ This behavior is characteristic of some ordered magnetic state; therefore we have built an Arrott plot (Figure 8b) that helps to prove the presence of the magnetic order and find the temperature of the phase transition. This is a well-established technique that requires a set of magnetization loops measured at different temperatures,^48−50^ and recently it was successfully used with XMCD magnetization curves as well.^51−53^ Mean field theory predicts that in the paramagnetic state a linear (high-field) part of the *M*^2^*vs H*/*M* plot, extrapolated to the low-field region, intercepts the positive part of the *H*/*M* axis, yielding the value of the inverse magnetic susceptibility. For the ferromagnetic state the crossing point at the *H*/*M* axis will be negative, while the isotherm, corresponding to the phase transition, passes through the origin (see also the discussion in the SI) .^48−50^Figure 8c shows positions of the interception *H*/*M*_*M*=0_ at different temperatures, obtained from the Arrott plot for the 0.6 ML sample. The negative intercept values found for all explored temperatures corroborate the existence of a magnetic order for the monolayer-thick NiBr_2_, which is the majority phase in this sample. The linear fit in Figure 8c results in a phase transition temperature of 27 K.

The existence of a noncollinear state agrees with theoretical calculations, which predict that a free-standing monolayer of NiBr_2_ is ordered ferromagnetically, but the competing interactions with the nearest and next nearest neighbors create a long-range helical magnetic texture.^41^ Experimentally, a helical magnetic structure with a periodicity of ∼40 unit cells was observed in the bulk NiBr_2_ at temperatures below 23 K.^38^ This noncollinear structure has zero magnetic moment if it is averaged over the region comparable in size to its periodicity. That would explain the absence of remanence in the monolayer-thick NiBr_2_, as observed in the 0.6 ML sample (Figure 7c,d). Nevertheless, we cannot exclude other types of noncollinear order, as proposed for 2D materials with in-plane magnetic anisotropy.^51,54^

### Mesoscopic Scale

**Figure 9:**
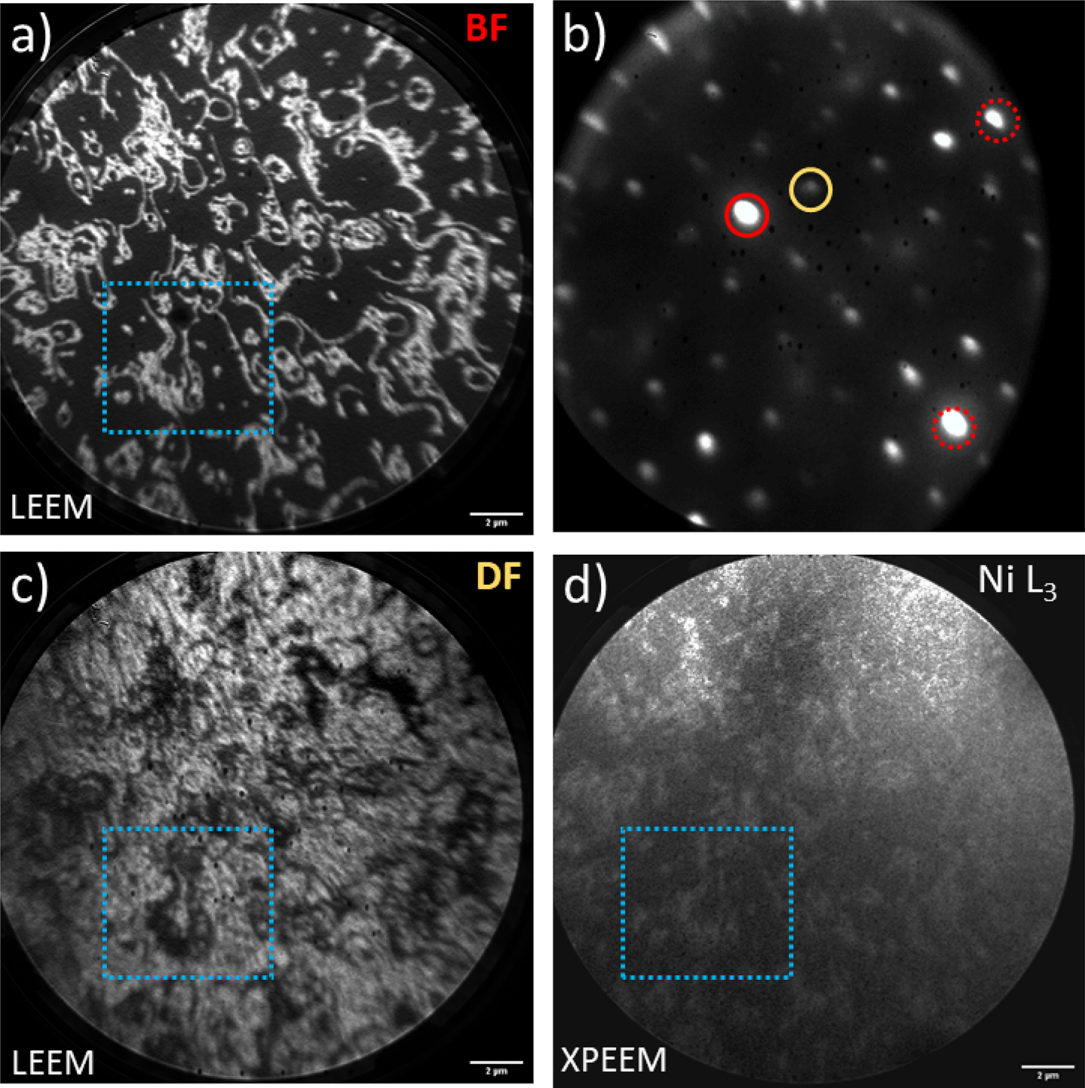
(a) Bright-field LEEM image of ∼1.5 ML NiBr_2_ grown on Au(111) at 400 K. (b) Corresponding LEED pattern, where the red circle highlights the (00) spot used for the bright-field images and the yellow circle indicates the spot characteristic of the 4 × 4 superstructure employed for the dark-field imaging. Dashed red circles show (10) and (01) spots that belong to the pattern of the NiBr_2_ hexagonal crystal structure. (c) Dark-field LEEM image. (d) XPEEM image showing the contrast in the absorption of the X-rays with the energy of the beam tuned to the peak of the L_3_ edge of Ni. All images were taken in the same area. Distinctive features repeated in all images are marked with the blue dotted rectangle.

Since the combined STS/XMCD measurements reveal a magnetic semiconducting behavior of NiBr_2_ down to the 2D limit, this material has an interesting potential to be used as a barrier in tunneling devices. It is thus important to study the growth modes and prove that film thickness is uniform within the length scale relevant for applications. For this aim, a sample of NiBr_2_ with ∼1.5 ML coverage was grown *in situ* in the preparation chamber of the CIRCE beamline (ALBA) and studied by means of LEEM and PEEM. A LEED pattern closely resembling the pattern shown in Figure 2c (that was measured in our own chamber for a sample with the same nominal coverage) demonstrated the presence of both NiBr_*x*_ and NiBr_2_ phases. Nevertheless, the LEEM image (not shown) acquired with a field of view of 10 μm showed no clear contrast, revealing the relatively small size of the crystalline domains, comparable to the resolution of the instrument. This observation corroborates the STM results, yielding as well a maximum island extension of 50–100 nm (see Figure 1). A more uniform growth was observed when the substrate was warmed up during deposition. Figure 9a shows a bright-field image, measured using the central (00) spot of the LEED pattern (Figure 9b) of a film with the same nominal amount of material but grown at 400 K. Micrometer-scale domains are clearly visible and were identified by dark-field LEEM (Figure 9c). In this mode a spot of the reconstruction pattern characteristic of the NiBr_*x*_ (Figure 1d) is used to build the image; therefore the domains of NiBr_*x*_ appear brighter than the domains of NiBr_2_.

Further, inspection with XPEEM shows a contrast in the X-ray absorption mode with a photon energy tuned to the peak of the Ni L_3_ edge (Figure 9d). Brighter zones correspond to the regions with stronger absorption (higher amount of Ni) and coincide with the domains of the NiBr_2_ phase. There are no macroscopic regions with distinctively different levels of absorption except these two, implying that a slab of NiBr_2_ uniformly grows on a continuous NiBr_*x*_ layer. Since a third layer of NiBr_2_ tends to cover the surface completely before the next layer starts to nucleate (see Figure 1c), we conclude that at least the first few layers of NiBr_2_ grow in a layer-by-layer mode, following the formation of a 1 ML thick wetting layer that contains NiBr_*x*_ and NiBr_2_ in a proportion depending on the substrate temperature.

## Conclusions

To summarize, we have demonstrated that the 2D NiBr_2_ compound grows epitaxially on Au(111) by means of sublimation of the stoichiometric precursor from a Knudsen cell. A combined LT-STM, LEED, and XPS study has unveiled that ultrathin 2D NiBr_2_ sheets on Au(111) tend to grow in a layer-by-layer mode. The first layer comprises two different phases: a stoichiometric NiBr_2_ layer and a NiBr_*x*_ compound with a commensurate crystalline structure and a distinctive reconstruction. The lower binding energy of Ni 2p states for the reconstructed phase seen with XPS together with a detailed analysis of the crystalline structure by LT-STM suggests that the NiBr_*x*_ phase corresponds to a partially dehalogenated NiBr_2_. Additionally, constant-current STS revealed a metallic behavior of this debrominated layer. The different layers of NiBr_2_ show layer-dependent characteristic d*I*/d*V* constant-current STS spectra, with a distinctive band gap for each layer and revealing strongly localized Ni-d states as predicted theoretically.

XMCD experiments have revealed noncollinear magnetic order with in-plane anisotropy in the NiBr_2_ down to the 2D limit. The critical temperature of the magnetic phase transition was found to be 27 K. At the same time, a monolayer-thick NiBr_*x*_ possesses simple ferromagnetic order with out-of-plane magnetic anisotropy below 40 K.

A mesoscopic scale characterization performed by means of LEEM and XPEEM confirmed a coexistence of small domains of both phases in the first monolayer if grown at room temperature. Warming the substrate to 400 K promotes a nucleation of NiBr_*x*_, leading to a continuous layer of this compound, while the next layers grow exclusively as stoichiometric NiBr_2_. Taking into account the easy preparation recipe with moderate technological requirements, high mechanical and electrical stability, and the large band gap down to the two-dimensional limit, we can foresee using NiBr_2_ for thin magnetic crystalline barriers in tunneling junctions and other electronic devices.

## Experimental and Methods

The Au(111) single crystal was cleaned in ultrahigh vacuum (UHV) by several sputtering–annealing cycles with an annealing temperature around 460 °C and kept at this temperature for 10 min. NiBr_2_ powder was sublimated from a Knudsen cell heated to 440 °C onto the clean Au(111) kept at room temperature, except one of the samples was prepared at 400 K for PEEM/LEEM characterization. The evaporation process of NiBr_2_ was monitored by a microbalance. The low-temperature STM experiments were carried out with an LT-STM (Createc GmbH) operated at 6 K under UHV conditions with a base pressure of 5 × 10^–11^ mbar. XPS and LEED experiments were performed in a separate UHV chamber (base pressure of 1 × 10^–10^ mbar). The XPS measurements were performed with a Phoibos 100 photoelectron spectrometer, using a non-monochromatic Al Kα X-ray source.

XMCD measurements were taken at two different beamlines at two Synchrotron Light Facilities: BOREAS beamline at ALBA and X-Treme beamline at SLS.^55,56^ Absorption spectra at the Ni L_2,3_ edge for both normal (θ = 0°, out of plane) and grazing (θ = 70°, almost in plane) incidence geometries were studied at different temperatures in a magnetic fields up to 6.5 T. For BOREAS experiments two samples were grown in our home laboratory with coverage close to 1 and 2 ML of NiBr_2_ and characterized by LEED and XPS prior to being transferred to the measurement chamber of the Boreas beamline, in a vacuum suitcase under a pressure below 1 × 10^–9^ mbar. In this way we can directly relate the XMCD signal with the sample structure and composition. The two sub-monolayer samples measured at the X-Treme beamline were grown *in situ* in the preparation chamber of the X-Treme beamline, which is equipped with an STM that allows establishing the direct relationship between the coverage and the integrated intensity of the polarization-averaged spectra (white line) listed in Table S1.

Mesoscopic-scale characterization was performed by means of LEEM/PEEM microscopy at the CIRCE beamline (ALBA).^57^ The samples were grown *in situ* in the preparation chamber. LEEM was employed to obtain bright- and dark-field images as well as microspot LEED patterns. In addition, XPEEM imaging at the Ni L_3_ edge was done.

## Supporting Information Available

FFT analysis of the NiBr_*x*_ phase; constant-height d*I*/d*V* spectra for bare Au(111) and NiBr_*x*_ and for the first and second layer of NiBr_2_; calculation of magnetic moment *via* sum rules analysis; Arrott plots for NiBr_*x*_; room-temperature STM and LEED data acquired at the Swiss Light Source (PDF)
