# Supplementary material for: Noncollinear Magnetic Order in Two-Dimensional NiBr2 Films Grown on Au(111)
Source: ACS Nano. 2021 Sep 7;15(9):14985–95. doi: 10.1021/acsnano.1c05221 (PMC8482757; doi:10.1021/acsnano.1c05221)
Supplement: Supplementary file 1 — nn1c05221_si_001.pdf [file nn1c05221_si_001.pdf]

Supporting Information: Non-Collinear Magnetic Order in Two-Dimensional $NiBr_2$ Films Grown on Au(111)

Djuro Bikaljević,[*,†,‡] Carmen González-Orellana,[¶] Marina Peña-Díaz,[¶] Dominik Steiner,[‡] Jan Dreiser,[§] Pierluigi Gargiani,[‖] Michael Foerster,[‖] Miguel Ángel Niño,[‖] Lucía Aballe,[‖] Sandra Ruiz-Gomez,[‖] Niklas Friedrich,[†] Jeremy Hieulle,[†] Li Jingcheng,[†] Maxim Ilyn,[*,¶] Celia Rogero,[¶,⊥] and José Ignacio Pascual[*,†,#]

†*CIC nanoGUNE-BRTA, 20018 Donostia-San Sebastián, Spain*

‡*Institute of Physical Chemistry, University of Innsbruck, Innrain 52c, A-6020 Innsbruck, Austria*

¶*Centro de Física de Materiales (CSIC/UPV-EHU), 20018 Donostia-San Sebastián, Spain*

§*Paul Scherrer Institut, Forschungsstrasse 111, CH-5232 Villigen PSI, Switzerland*

‖*ALBA synchrotron Light Source, Carrer de la Llum, 2-26 08290 Barcelona, Spain*

⊥*Donostia International Physics Center DIPC, 20018 Donostia-San Sebastián, Spain*

#*Ikerbasque, Basque Foundation for Science, 48013 Bilbao, Spain*

* E-mail: djuro.bikaljevic@uibk.ac.at; maxim.ilin@ehu.es; ji.pascual@nanogune.eu

September 1, 2021

Fast-Fourier transformation (FFT) analysis of the $NiBr_x$ phase

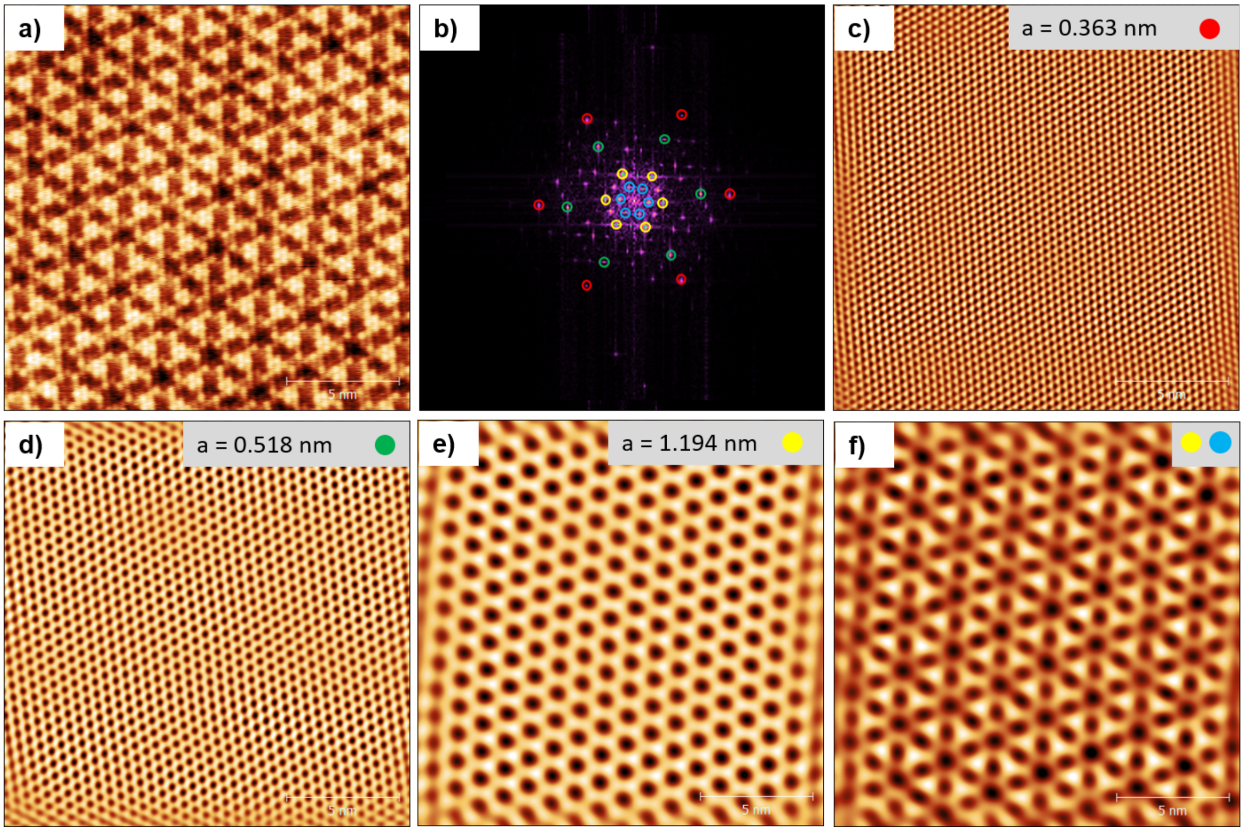

Figure S1: (a) Atomic layer structure of $NiBr_x$(green rectangle). I = 20pA, U = 1V. (b) Fourier transformed image of (a). (c-f) fractional images of (a).

Fig. S1 shows the FFT analysis for the $NiBr_x$ phase as already reported in Fig. 1 of the main manuscript. It contains four sets of hexagonal patterns, where the inner hexagons (indicated by the green, yellow and blue circles) are slightly rotated relative to the mirror axis that is defined through two red circles with respect to the outmost hexagon, eventually suggesting a chiral structure of $NiBr_x$. The periodicity of the largest hexagon (marked with red circles in Fig. S1b) equals to ≈ 3.6 Å, presumably corresponding to a commensurate Ni-plane on Au(111). This value is close to the predicted lattice constant of $NiBr_2$ sheets of 3.7 Å and clearly differs with respect to the lattice constant of Au(111) ($a_{\mathrm{Au(111)}} \approx 2.87\AA$). It is worth to say that with exception of the rotation of the two inner hexagons in the FFT the LEED pattern for $NiBr_x$ in Fig. 2b resembles very much. Since the LEED image was taken at room-temperature and the STM at 7 K, the chirality in the STM image may be consequence of a phase transition of the $NiBr_x$ phase, taking place at low temperatures. This assumption is further substantiated by room-temperature STM data (see Fig. S7) where for

the $NiBr_x$ phase no chirality can be observed but showing as well the same super periodical structure of ≈ 11 Å.

Constant height dI/dV

Fig. S2 shows two spectra, in yellow the constant height dI/dV signal for Au(111) and in blue the dI/dV signal measured on $NiBr_x$. Both spectra were measured with the same Lock-In parameters. As expected, the bare Au(111) surface clearly shows the existence of the Au(111) Shockley surface state at $\approx$ -500 mV. Upon deposition of $NiBr_x$ the Au(111) surface state is quenched, suggesting a strong interaction of the $NiBr_x$ with the metal substrate.

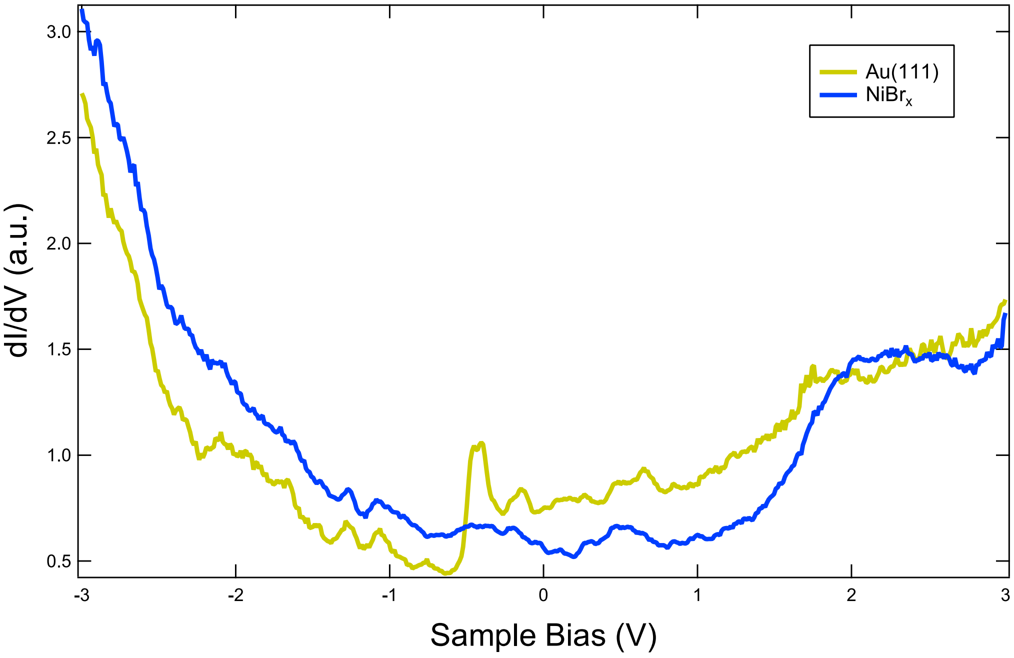

Figure S2: Feedback controlled dI/dV spectrum for Au(111) (yellow) and of $NiBr_x$ (blue). Lock-In parameters: current set-point: 1 nA

In Fig. S3 a full-range constant height dI/dV spectrum for the 1st and 2nd slab of $NiBr_2$ is displayed, demonstrating a depleted dI/dV signal within the determined effective gap as described in Fig. 5. The onset of the valence band (VB) for both layers can be estimated to $\approx$ -2.3 ($\pm$ 0.2) V. Regarding the onset of the conduction band (CB), a clear shift of the peak position between the black and red spectrum is observed. As already described in section "Layer dependent electronic band gap" (Fig. 4 and Fig. 5) of the main manuscript, the electrostatic shift of the localized Ni-d state is explained due to screening effects in response to the applied sample-tip potential. At first glance the Au(111) Shockley surface state (at $\approx$ -500 mV) seems to be lost upon $NiBr_2$ deposition. However, we cannot rule out, that the

Au(111) surface state vanishes completely, especially when looking at the black spectrum in Fig. S3.

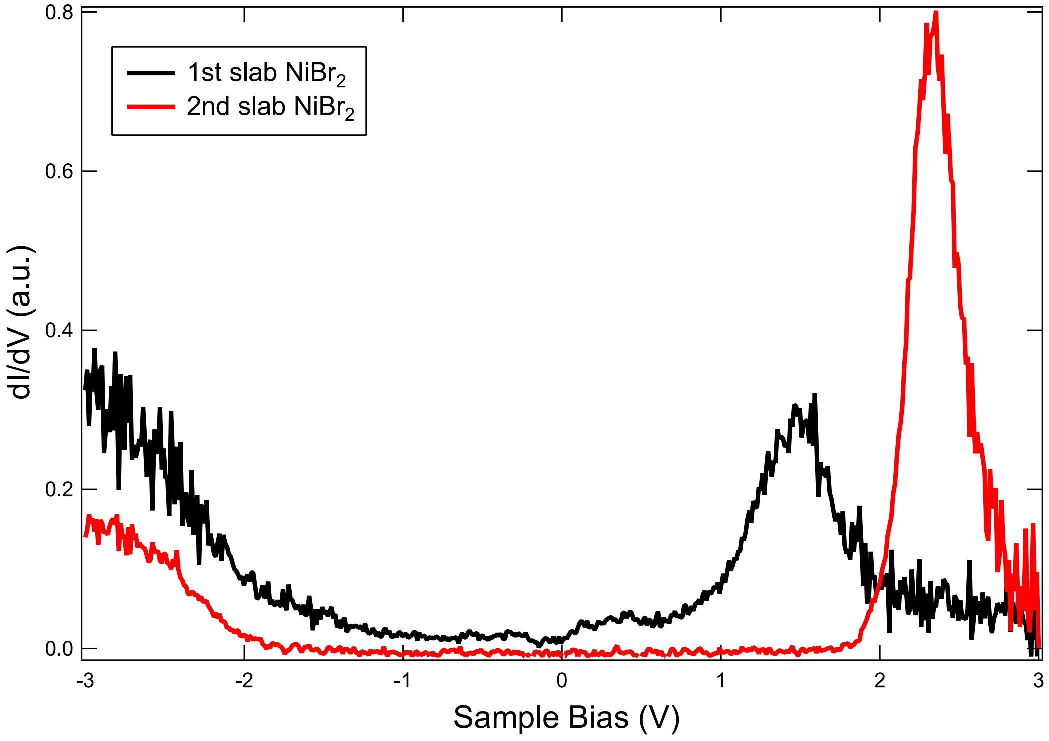

Figure S3: Feedback controlled dI/dV spectrum for the 1st (black) and 2nd layer (red) of $NiBr_2$. Lock-In parameters (black and red): modulation voltage: 20mV, f = 760 Hz, current set-point: 100 pA

Calculation of magnetic moment *via* sum rules analysis

The magnetic moment of the samples was calculated applying the sum rules to the XMCD data. We use Chen's notation,[1] so in the case of $L_{3,2}$ edges, the orbital (m_{orb}) and spin (m_{spin}) magnetic moments are related to the p, q and r parameters as follows:

$$m_{orb} = -\frac{2q}{3r}N_h \tag{1}$$

$$m_{spin} = -\frac{3p-2q}{r}N_h\left(1+\frac{7T_z}{2S_z}\right)^{-1} \tag{2}$$

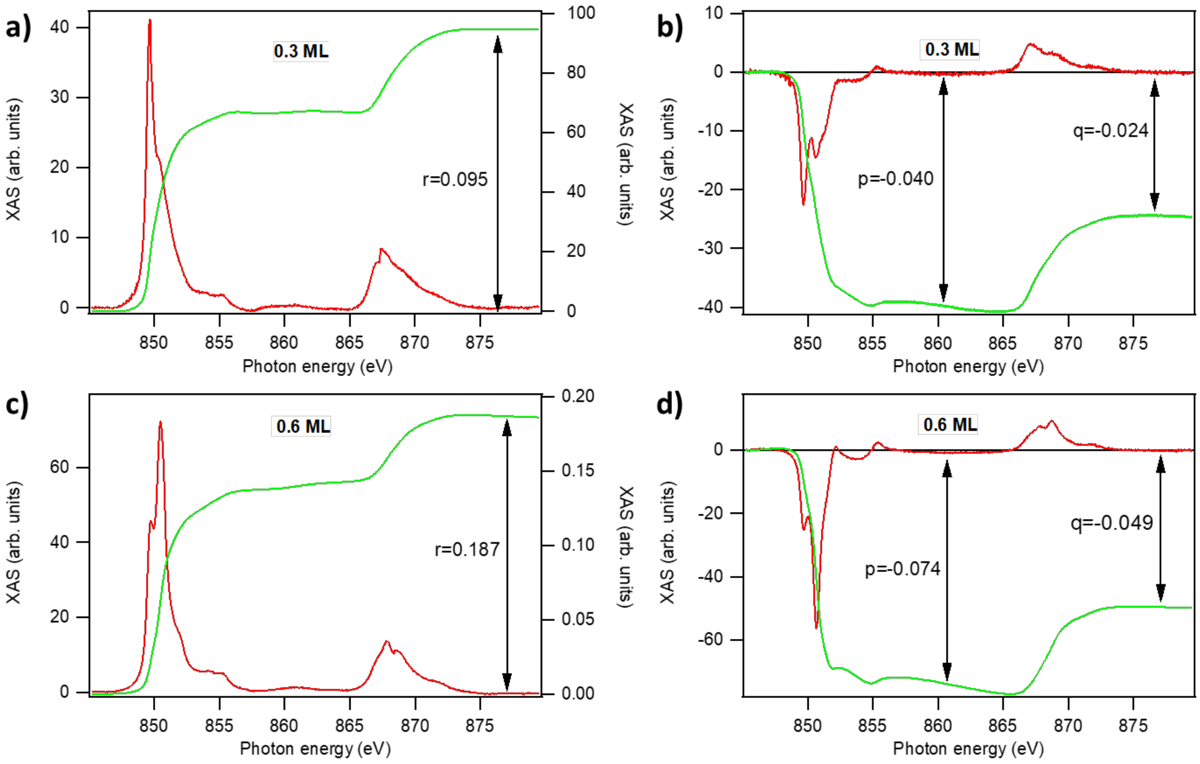

Figure S4: X-ray Magnetic Circular Dichroism, XMCD (right column) and white line of absorption spectra measured at the Ni L_{32} edge in grazing incidence at 3 K in the field of 6 T. The p, q and r parameters shown in the figure are the three integrals necessary for sum-rules analysis.

N_h is the number of holes in the probed state, whereas T_z and S_z are the expectation values of the spin magnetic dipole and spin operators, respectively. For these calculations N_h was taken to be equal 2, supposing that Ni has two holes in the d-shell. Furthermore, a contribution of the dipole term was neglected since it is smaller than 10 % for Ni^{2+} in the octahedral coordination.[2] Figures S4 and S5 illustrate application of the sum rules and the values of the calculated moments are listed in the Table S1. A noise level in the experimental spectra is negligibly low even for the samples with only 0.3 ML of $NiBr_x$, therefore the major error in the calculated values of the moments is concerned with subtraction of the background before the integration. This uncertainty leads to non-constant values of the integrated spectra $p(E)$, $q(E)$ and $r(E)$ between the L_3 and L_2 absorption edges, as well as after the L_2 edge. Taking this deviation from a constant value as an estimation of the error we can roughly calculate the overall precision of the moments in the Table S1 as 10 %.

Since the samples with a coverage of 0.3–1.0 ML have two different phases containing Ni ions some precautions have to be taken. Both equations are still valid, since the integration is a linear operation and the integral of the sum of two functions is a sum of integrals of each function taken separately, but a different number of holes N_h can be an issue. It is easy to demonstrate for the orbital moment sum rule eq.1, although the same calculations can

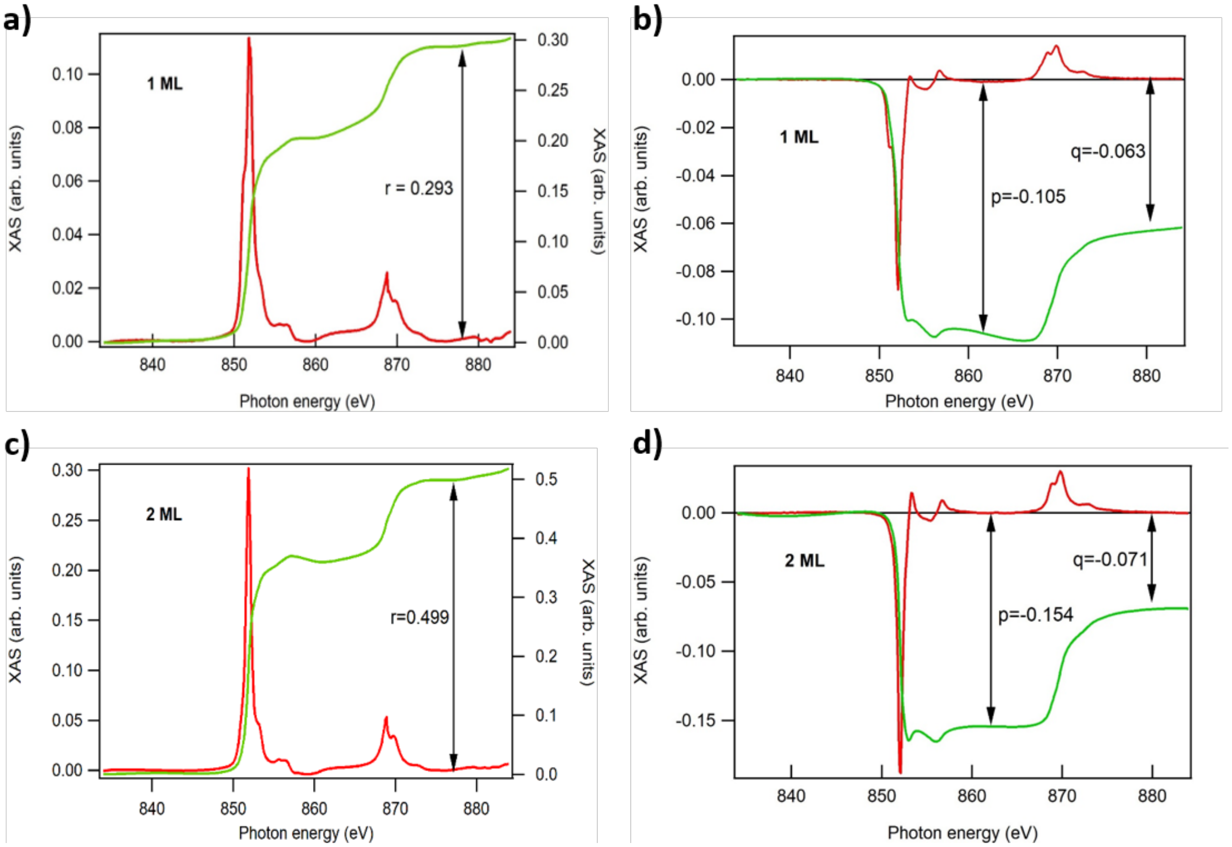

Figure S5: X-ray Magnetic Circular Dichroism, XMCD (right column) and white line of absorption spectra measured at the Ni L_{32} edge in grazing incidence at 3 K in the field of 6 T.

be easily done for the spin sum rule eq.2. Indeed, if we assign f_i and F_i for the difference (XMCD) and for the polarization-averaged spectra, the orbital moments of each phase is represented as:

$$\frac{m_{orb1}}{N_h} = -\frac{2q_1}{3r_1}, \qquad \frac{m_{orb2}}{N_h} = -\frac{2q_2}{3r_2} \tag{3}$$

where

$$q_i = \int f_i(E)dE \quad and \quad r_i = \int F_i(E)dE. \tag{4}$$

Then, the weighted average of the orbital moments is

$$\frac{r_1}{r_1+r_2}\frac{m_{orb1}}{N_h} + \frac{r_2}{r_1+r_2}\frac{m_{orb1}}{N_h} = -\frac{r_1}{r_1+r_2}\frac{2q_1}{3r_1} - \frac{r_2}{r_1+r_2}\frac{2q_2}{3r_2} = -\frac{2(q_1+q_2)}{3(r_1+r_2)} \tag{5}$$

where both numerator and denominator are the integrals of the sum of the spectra:

$$
\begin{aligned}
q_1 + q_2 &= \int f_1(E)dE + \int f_2(E)dE = \int f_1(E) + f_2(E)dE = q \\
r_1 + r_2 &= \int F_1(E)dE + \int F_2(E)dE = \int F_1(E) + F_2(E)dE = r
\end{aligned}
\tag{6}
$$

This result is precise and it is of frequent use in the systems of mixed-valence compounds,[3,4] but it requires the same value of N_h for both phases if we want to keep the equations 1-2. Otherwise, the non-integer effective value of N_h has to be used.[3,4] In our case we do not know the number of holes per Ni ion in the $NiBr_x$. But, a relatively weak shift of Ni components in the XPS spectra (Fig. 3) implies rather a variation of the coordination than a different valence. Therefore, we assume that N_h of the $NiBr_x$ is the same as in the stoichiometric $NiBr_2$.

Table S1: Magnetic moments calculated from the XMCD data by sum rules. Estimated value of the experimental error is 10%.

Sample	Temp. (K)	Orientation	m_{orb}(μ_B/Ni at)	m_{spin} (μ_B/Ni at)	m (μ_B/Ni at)	White line (r)
0.3 ML	3	GI	0.34	1.45	1.79	0.10
	3	NI	0.38	1.28	1.66	0.11
0.6 ML	3	GI	0.36	1.27	1.63	0.19
	3	NI	0.41	1.49	1.90	0.17
1 ML	3.5	GI	0.36	1.28	1.64	0.29
2 ML	3.5	GI	0.11	1.47	1.58	0.5

Arrott plots

This technique is based on the power series expansion of the thermodynamic potential close to the phase transition temperature. It was shown analytically that a temperature-dependent factor of the M^2 term in the expansion has to become zero at the transition point, being negative in the ordered state and positive in the paramagnetic state. This factor is equal to the zero-field inverse magnetic susceptibility and can be obtained from a linear fit of the high-field part of the M^2 *vs* H/M plot, constructed from experimental magnetization loops for various temperatures.[5,6] Equivalent treatment within the mean field theory predicts that in the paramagnetic state a linear (high-field) part of the M^2 *vs* H/M plot, extrapolated to the low-field region, intercepts the positive part of the H/M axis, yielding the value of the inverse magnetic susceptibility. For the ferromagnetic state the crossing point at the H/M axis will be negative, while the isotherm, corresponding to the phase transition, passes through the origin.[7] Fig. S6a shows a set of the XMCD magnetization curves for 0.3 ML sample, respective Arrott plot (Fig. S6b) and positions of the interception $H/M_{M=0}$ at different temperatures. The negative intercept values prove the existence of the magnetic order in the sample and a linear fit of the $H/M_{M=0}$ *vs* T data yields 40 K as an estimation of the Curie temperature for the monolayer-thick $NiBr_x$, which is the majority phase in this sample. Modified Arrott plot technique could be also used to find the critical exponents fitting $M^{1/\beta}$ *vs* $(H/M)^\gamma$ but a presence of two phases with different Curie temperatures would undermine the precision of these data .[8]

Although the XMCD magnetization loops acquired in the field-sweep mode do not allow reliable measurements within approximately ±0.1 T and therefore do not show hysteresis due to magnetization reversal of the $NiBr_x$, there are more evidences of the ferromagnetic order in this sample. It was mentioned in the section Magnetic Properties that magnetization remains constant down to the lowest measurable fields for the NI geometry (see figure 7b) and it implies non-zero remanence. Actually, magnetization at zero internal field (remanence) can be estimated more precisely from our experimental data. Since $NiBr_x$ has an out-of-plane magnetic anisotropy, internal magnetic field is lower than applied field by demagnetization field of the infinite plane $4\pi M$.[6] Magnetization M of $NiBr_x$ can be estimated using magnetic moment $m = 1.7\mu_B$ of Ni atom for the 0.3 ML sample listed in the Table S1 and the average atomic volume of the $NiBr_2$ structure[9] $v \approx 24\mathring{A}^3$ (figure 1 shows similar hexagonal arrangement and atomic periodicity of $NiBr_x$ and $NiBr_2$). With these assumptions demagnetization field can be calculated as:

$$4\pi M = \frac{4\pi m}{3v} = \frac{12 \cdot 1.7 \cdot 9.2 \cdot 10^{-21}}{3 \cdot 24 \cdot 10^{-24}} \approx 2.6 \cdot 10^3 \ (G) = 0.26 \ (T) \tag{7}$$

It means that internal field is zero when external field is lower than 0.26 T and therefore remanence is equal to the magnetization at 0.26 T, which is clearly visible in the figure 7b. Furthermore, it is easy to demonstrate that non-zero remanence does not originate from

magnetic anisotropy as, for example, in the molecular magnets. Indeed, figure 7a allows us to estimate the value of field that saturates the magnetization in the hard magnetization direction $H_A \approx 2\ T$ (so-called anisotropy field). With this value, the anisotropy energy E_A per Ni atom can be found as:[6]

$$E_A = \frac{m \cdot H_A}{2} = \frac{1.7 \cdot 5.8 \cdot 10^{-5} \cdot 2}{2} \approx 9.9 \cdot 10^{-5}\ (eV) \approx 0.1\ (meV) \tag{8}$$

Then, using a definition $25 = E_A/k_B \cdot T_B$,[5] the superparamagnetic blocking temperature per Ni atom is:

$$T_B = \frac{E_A}{25 \cdot k_B} = \frac{1 \cdot 10^{-4}}{25 \cdot 8.6 \cdot 10^{-5}} \approx \frac{1}{20}\ (K) = 0.05\ (K) \tag{9}$$

This value is much lower than the temperature of measurements and therefore magnetic anisotropy is too weak to provide a non-zero remanence observed in the figure 7b. In summary, a monolayer-thick islands of $NiBr_x$ that comprise a majority phase of the 0.3 ML sample, possess at 3K a non-zero magnetic moment in zero internal magnetic field that does not increase further when the external field up to 6 T is applied along the out-of-plane direction. Furthermore, this non-zero moment is not stabilized by magnetic anisotropy. This behaviour is characteristic of the ferromagnetic ordered state, which corroborates the conclusion driven from the analysis of the Arrott plot (Fig. S6b).

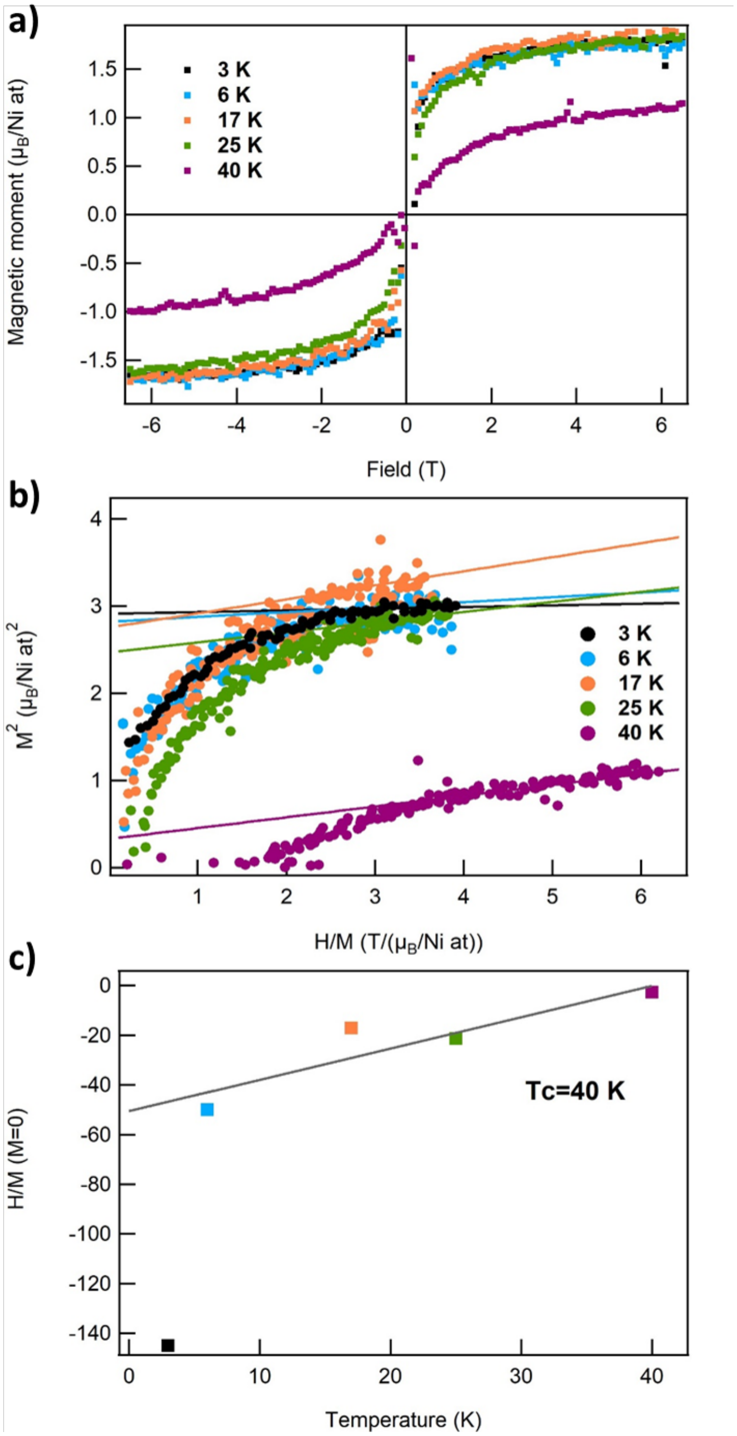

Figure S6: Arrott plot made for the 0.3 ML sample, having majority of single layer thick $NiBr_x$ phase.

Room-temperature STM and LEED data acquired at the Swiss-Light-Source

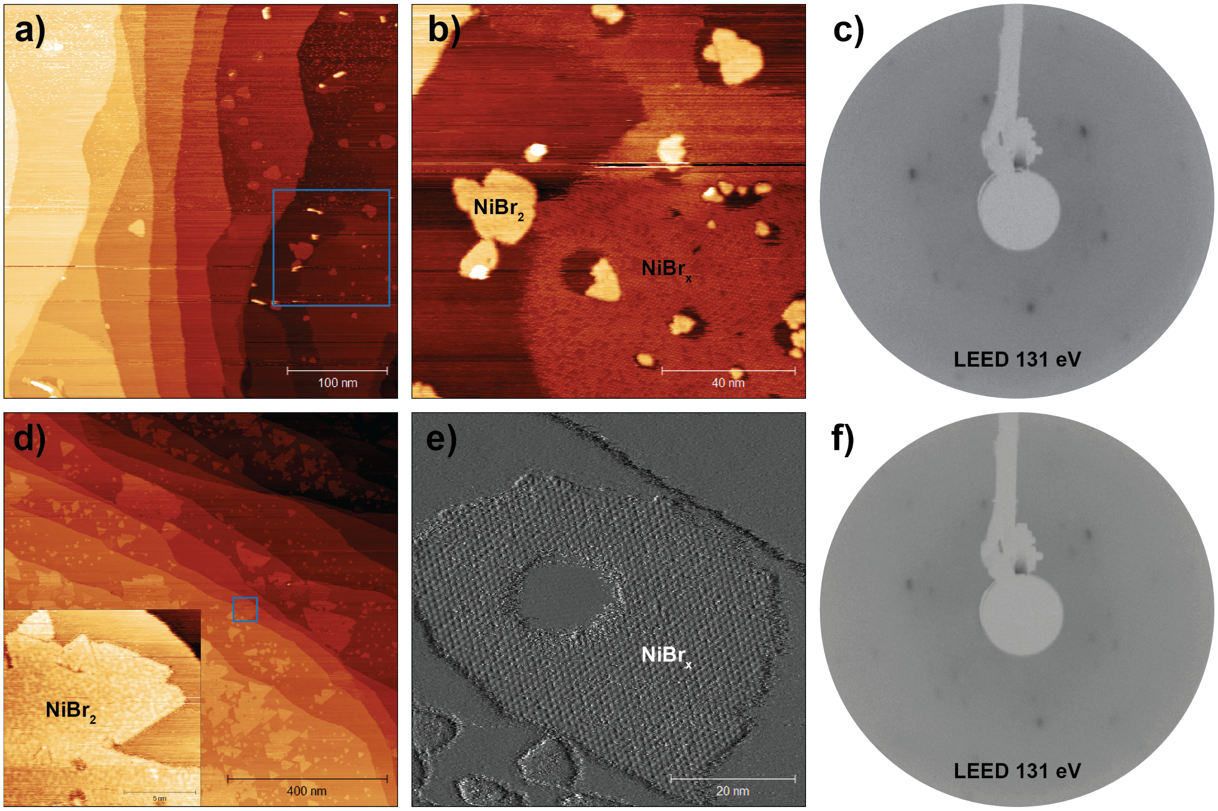

Figure S7: Room-temperature STM images with the corresponding LEED pattern of two different preparations of $NiBr_2$ on Au(111). 1st preparation (a-c), showing fractionally more of the $NiBr_x$ species, next to islands of $NiBr_2$. 2nd preparation (d-f) showing fractionally more pristine $NiBr_2$ on the Au(111) surface. Different fractions of the two species are obtained with different deposition rates during the evaporation process. Blue rectangles in (a) and (d) are displayed enlarged in (b) and (e), respectively. Inset in (d) reveals the $NiBr_2$ phase in more detail, showing the morphology reported in Fig. 1. (e) constant height image of the $NiBr_x$ phase at room-temperature revealing a periodicity for the $NiBr_x$ phase of ≈ 11 Å. Tunneling parameters: (a) I = 50pA, U = 2.1V; (b) I = 50pA, U = 2.1V; (d) I = 1.5nA, U = 1.0V; inset in (d) I = 1.8nA, U = 75mV; (e) I = 1.5nA, U = 1V.

Room-temperature STM was used to obtain an estimation of the $NiBr_2$ coverage on Au(111) for the subsequent XAS and XMCD experiments, measured at the X-Treme beamline at the Swiss Light Source (Paul Scherrer Institute). Fig. S7 shows room-temperature STM data and the corresponding LEED pattern for two submonolayer preparations of $NiBr_2$ on Au(111) acquired at different evaporation rates. In the 1st preparation (≈ 0.3 ML, Fig. S7a,b) a coexistence of $NiBr_2$ and $NiBr_x$ can be seen, where the latter one is predominately

present at the surface. The corresponding LEED pattern in Fig. S7c shows the existence of the superstructure, as already described in Fig. 1 of the main manuscript. The second preparation with an estimated coverage of ≈ 0.6 ML shows predominantly the proper $NiBr_2$ phase, with small amounts of the decomposed $NiBr_x$ phase (Fig. S7d). The inset in Fig. S7d shows a zoom-in of a $NiBr_2$ island, revealing the same bright and dark regions in the 1st layer as in Fig. 1b of the main manuscript. Fig. S7e demonstrates a constant current image of the $NiBr_x$ phase, exhibiting a periodicity of ≈ 11 Å and thus in line with low-temperature STM data (see Fig. 1). The corresponding LEED image is displayed in Fig. S7f.

References

1. Chen, C. T.; Idzerda, Y. U.; Lin, H.-J.; Smith, N. V.; Meigs, G.; Chaban, E.; Ho, G. H.; Pellegrin, E.; Sette, F. Experimental Confirmation of the X-Ray Magnetic Circular Dichroism Sum Rules for Iron and Cobalt. *Phys. Rev. Lett.* **1995**, *75*, 152–155.

2. Teramura, Y.; Tanaka, A.; Jo, T. Effect of Coulomb Interaction on the X-Ray Magnetic Circular Dichroism Spin Sum Rule in 3D Transition Elements. *Journal of the Physical Society of Japan* **1996**, *65*, 1053–1055.

3. Heidler, J.; Piamonteze, C.; Chopdekar, R. V.; Uribe-Laverde, M. A.; Alberca, A.; Buzzi, M.; Uldry, A.; Delley, B.; Bernhard, C.; Nolting, F. Manipulating Magnetism in $La_{0.7}Sr_{0.3}MnO_3$ *via* Piezostrain. *Phys. Rev. B* **2015**, *91*, 024406.

4. Koide, T.; Miyauchi, H.; Okamoto, J.; Shidara, T.; Sekine, T.; Saitoh, T.; Fujimori, A.; Fukutani, H.; Takano, M.; Takeda, Y. Close Correlation between the Magnetic Moments, Lattice Distortions, and Hybridization in $LaMnO_3$ and $La_{1-x}Sr_xMnO_{3+\delta}$: Doping-Dependent Magnetic Circular X-Ray Dichroism Study. *Phys. Rev. Lett.* **2001**, *87*, 246404.

5. Ionescu, A.; Llandro, J.; Ziebeck, K. R. A. In *Magnetic Nanoparticles in Biosensing and Medicine*; Darton, N. J., Ionescu, A., Llandro, J., Eds.; Cambridge University Press, UK, 2019; p 1–51.

6. O'Hendley, R. C. Modern Magnetic Materials:Principles and Applications. *Johns Wiley & Sons, Inc., New York, 2000*

7. Arrott, A. Criterion for Ferromagnetism from Observations of Magnetic Isotherms. *Phys. Rev.* **1957**, *108*, 1394–1396.

8. Arrott, A.; Noakes, J. E. Approximate Equation of State for Nickel near Its Critical Temperature. *Phys. Rev. Lett.* **1967**, *19*, 786–789.

9. Rai, B. K.; Christianson, A. D.; Mandrus, D.; May, A. F. Influence of Cobalt Substitution on the Magnetism of NiBr2. *Physical Review Materials* **2019**, *3*, 034005.
